# Tropical fruits as a potential source for the recovery of bioactive compounds: *Tamarindus indica L., Annona muricata, Psidium guajava* and* Mangifera indica*

**DOI:** 10.1007/s13197-024-05983-5

**Published:** 2024-05-22

**Authors:** Mashell A. Toscano Oviedo, Luis A. García Zapateiro, Somaris E. Quintana

**Affiliations:** Research Group on Complex Fluid Engineering and Food Rheology (IFCRA), University of Cartagena, 130015 Cartagena, Colombia

**Keywords:** Bioactive compounds, Tropical fruits, Healthy foods, Biological activities

## Abstract

The objective of this review is to identify the bioactive compounds present in tropical fruits such as *Tamarindus indica L., Annona muricata, Mangifera indica*, and *Psidium guajava* and their biological activities. The identification of these compounds shows their potential as a food ingredient in the development of products, providing added value to them, because not only the pulp of the fruit is used, but also the shell and its other parts, such as the leaves, are used, being viable sources to obtain some compounds that benefit human health. Implementing fruits that have certain bioactive compounds such as carotenoids (β-carotene, α-carotene, lutein, zeaxanthin and β-cryptoxanthin), antioxidants (vitamins A and C), and phenolic compounds (ellagic acid, gallic acid, citric acid) in the production process in the food industry, allows them to become functional foodstuffs. The results obtained show the need to implement the operational processes that allow obtaining different compounds, which ensure their stability and precision, applying different extraction methods such as maceration, Soxhlet, supercritical fluids, and ultrasound.

## Introduction

Today, the demand for healthy food intake is increasing; different investigations support diets rich in compounds with biological activities that significantly reduce some diseases, allowing health benefits if they are accompanied by a healthy lifestyle (Swinburn et al. 2015).

The acquisition of healthy food for a profitable life presents a challenge considering cultural foods and environmental factors that promote the demand for these products. Each food generates its value, which demonstrates the essential benefit for people (Plasek et al. 2020); fruits and vegetables have components such as vitamins and minerals, dietary fiber and compounds with antioxidant capacity such as vitamin C, β-carotenes, carotenoids, and flavonoids that exposed functional and beneficial properties (Prada et al. 2007). Fruits are presented as food with very favorable specific nutritional components and used among integrated medicines as alternative therapies for the reduction of basic diseases, which radically affect organisms. Tropical fruits are gaining popularity all over the world due to their taste and nutritional value and the significant contribution of macro and micronutrients; this is reflected as bioactive substances that, despite not having a real nutritional role in the body, can regulate certain physiological functions in the body (Jiménez-Colmenero 2013), in addition to impacting the treatment of some diseases, which causes great interest in the field of research (Hannum 2004).

Plants and vegetables are indeed known to possess a high content of compounds that can capture and neutralize free radicals such as carotenoids, polyphenols andanthocyanins, making them usable in prophylactic and curative phytotherapy. Consequently, four large groups of bioactive compounds can be distinguished, these are nitrogenous, sulfur, terpenic, and phenolic substances; where, the latter have been the subject of study in recent years (Tomás-Barberán 2003). Now, within these groups, there are thousands of components, which are identified by their unique presence in the plant kingdom and are usually grouped in foods, so that on certain occasions a certain substance is located in a group or vegetable family. For this reason, the study of each of these fruits as a potential raw material for the development of healthy foods is essential to examine the details of their benefits in organisms and to look for the outstanding and functional aspects of the fruits in question and their use. Therefore, the objective of this review is to identify the bioactive compounds present in T*amarindus indica L., Annona muricata, Mangifera indica, and Psidium guajava* and their biological activities.

## Chemical constituent and biological activities

Bioactive compounds are considered secondary metabolites without nutritional function, whose origin is vegetable, but they are of great importance in the functioning of the human organism. These are included in diets since, in addition to providing their biological effects, they improve the sensory characteristics of foods. This caused some interest to be generated by the scientific community and consumers in general (Ramful et al. 2011) in the study of these compounds as food ingredients. In tropical fruits, the presence of these compounds is subject to the type of fruit and it is a morphological part, since, in the case of carotenoids, they have a greater presence on the surface of the tissues, but on the other hand, the phenolic compounds such as flavonoids, tannins, and lignins, they are located especially in the shell, seeds and in some cases in the pulp (Ayala-Zavala et al. 2011).

### Tamarindus indica L.

*Tamarindus indica L.* is cultivated in tropical regions, Asia, Africa, America, and many Caribbean islands; the fruit has a low water content and a high content of carbohydrates, protein, and minerals compared to other fruits (Bravo Aranibar and Bravo Araniba 2016). Then, different bioactive compounds have been determined in the plant: The pulp has different types of organic acids such as tartaric, citric, and malic acid; leaves present carotenoids such as β-carotene, α-carotene, lutein, zeaxanthin, and β-cryptoxanthin (Eldahshan et al. 2013); and the roots present alkaloids, tannins, steroids, and triterpenoids (Gupta and Singh 2017). The content of bioactive compounds is associated with biological activities; the root has been used in different applications that range from studies in medicine to as a fundamental component in the food industry; however, the works specifying the functional and nutritional properties of this fruit are limited (Páez-Peñuñuri et al. 2015), then roots have been associated with enhanced pain inhibition (Gupta and Singh 2017); leaves promote immune system enhancement, also serve as tissue photoprotection and have a high antioxidant capacity, which is also due to the existence of other compounds such as phenolics (Eldahshan et al. 2013), which can act as antioxidants and antimicrobials (Balbuena-Escalona 2012); the pulp has antioxidant activity due to high concentrations of polyphenolic compounds (Granados-Conde et al. 2017).

The identification of bioactive compounds from *Tamarindus indica L.* shows its potential as a food ingredient in the development of products, providing added value to them; because not only the pulp of the fruit is used, but the shell and its other parts, such as the leaves, are also used, being viable sources to obtain some compounds that benefit human health. The extracts from fruits present volatile compounds such as $\alpha$-pinene, $\beta$-pinene, myrcene, furfural, linalool, carvacrol, $\alpha$-copaene, $\beta$-caryophyllene, terpinene-4-ol, $\alpha$-humulene, $\gamma -$ cadinene, octadecane; Fagbemi et al. (2021) obtain extracts fractions from seed employee soxhlet extraction with p-Xylene, Hydrazinecarboxylic acid, phenylmethyl, Benzeneethanol, alpha-, beta-dimethyl-, 1-Decyne, 1-Hexanol, 2-ethyl-, Oxalic acid, isobutyl nonyl ester, p-Cymene, *n*-Hexadecanoic acid, Tritetracontane; Fagbemi et al. (2021) obtain essential oil from seed with o-Xylene, p-Xylene, 3-tert-Butyl-5-chloro-2-hydroxy benzophenone, Dodecane, 2H-pyran, tetrahydro-4-methyl-2-(2-methyl, cis-3-Methylcyclohexanol, Benzene, 1-ethyl-2,3-dimethyl, Undecane, n-Hexadecanoic acid, Phytol and acetate; while Cvetanović et al. (2020) obtain extracts from seeds with bioactive compounds such as epicatechin, rutin, quercetin-O-hexoside, hydroxybenzyl-hexose-pentose, protocatechuic acid and chlorogenic acid, and antioxidant activity.

### Annona muricata

*Annona muricata* is a plant that is cultivated from Central America to South America. The fruit has different minerals such as K, Ca, Na, Cu, Fe, and Mg, which contribute to the supply of nutrients and necessary elements for the body (Gyamfi et al. 2011). Different parts of *Annona muricata* present bioactive compounds. The fruits present isomers of annonacinone, isomers of gonionenin, isomers of montanacin-D (Laboureur et al. 2017), leaves ppresent12,15-cis-squamostatin-A, squamostatin-A; bullatacin; Squamocin; Isodesacetyluvaricin; Desacetyluvarici (Nik Mat Daud et al. 2015) and by-products. Gallic acid, Coumaric acid, Cinnamic acid, Caffeic acid, Chlorogenic acid, Protocateic acid, Hydroxybenzoic acid, Syringic acid, and Neochlorogenic acid (Aguilar-Hernández et al. 2019).

The applications of this plant and the fruit are natural medicine, and this has made use of all the morphological parts that comprise it, including bark, leaves, roots, and fruits, although studies have shown that the highest concentration of active compounds is found in leaves (Coria-Téllez et al. 2018). At a general level, the fruit is the most studied because of its application in the food industry. In this way, *Annona muricata* is considered a medicinal plant that works as an easily accessible alternative to the treatment of gastric and gastrointestinal cancer in many countries of the world (Alonso-Castro et al. 2011).

*Annona muricata* leaf extracts have been shown to have antioxidant properties (Nandhakumar and Indumathi 2013) within which there are various compounds such as vitamins C and E, carotenoids, anthocyanins, flavonoids, and other phenolic compounds correlated with their antioxidant capacity (Contreras-Calderón et al. 2011); Furthermore, it is also related to the presence of compounds such as acetogenins and alkaloids, where it has been verified that acetogenins induce death by inhibition of mitochondrial complex I in hepatocellular carcinoma cells (HepG2) (de Pedro et al. 2013). Then, Olivier et al. (2014) obtained total polyphenols in a dry form from Annona muricata leaves showing values of 39.57 ± 0.043 mg/100 g, carotenoids 0.28 ± 0.033 mg/100 g, and vitamin C 33.24 ± 0.160 mg/100 g. On the other hand Kuskoski et al. (2005) carried out Brazil the quantification of total phenols in frozen soursop pulp, obtaining values of 84.3 ± 5.8 mg GAE/100 g, in addition to the fact that other values obtained in soursop pulp are recorded in terms of vitamin C content, reported by Isabelle et al. (2010), the amount of this was 15.98 mg AA/100 g, while Ogunlesi et al. (2010) obtained these values in the pulp by two methods, obtaining as a result concentrations of 13.63 and 10.51 mg AA/100 g.

*Annona muricata* has a certain bioactive compound, such as vitamin C, which is present in higher concentrations in the pulp. The soursop pulp is used to make nectar, desserts, and other products; however, it is important to note that parts such as the leaves, seeds, and roots of this plant have significant concentrations of bioactive compounds, making them potential material for the development of different products in different industries.

### Mangifera indica

*Mangifera indica* is a fruit tree species that belongs to the genus Mangifera, which encompasses approximately 30 species that have different medicinal properties in the various morphological parts of the tree (Yahia 2011). The fruit is generally consumed fresh worldwide since it has remarkable sensory and nutritional characteristics such as vitamins, fiber, phytochemicals, and minerals of which potassium and magnesium are highlighted, these participate in nerve and muscle transmission, in addition, it provides small portions of other minerals such as iron, phosphorus, and calcium (Ramírez and Pacheco de Delahaye 2011).

This plant has not been thoroughly studied, so its full potential is uncertain, despite having been shown to be parts such as the peel, the seed and the pulp, which are generally not used, are suitable materials to obtain extracts rich in phenolic compounds with antioxidant characteristics; additionally, an environmental benefit can be obtained through its use, i.e. Sumaya-Martínez et al. (2019), since with the matter resulting from the pulping of the ataulfo cultivar of Mangifera indica, it was determined that it had a high antioxidant activity and a high concentration of bioactive compounds; therefore phenolics appear as the main constituents.

Serna-Cock et al. (2015). studied the main antioxidant compounds present in the freeze-dried powders of mango peels, for this, they considered 3 varieties of mango, Criollo (sugar mango), Keitt, and Tommy Atkins, which in each case showed significant differences, since the Creole variety obtained the following results: in anthocyanins 33.275 ± 6.07, carotenoids 17.623 ± 0.77, lycopene 1.837 ± 0.14 and ascorbic acid 512.637 ± 59.97. For the Keitt variety, the results for each case were lycopene 1.946 ± 0.12, carotenoids 19.200 ± 1.36, anthocyanins 16.026 ± 0.73, and ascorbic acid 338.095 ± 21.48. Finally, the Tommy Atkins variety for ascorbic acid 332.967 ± 28.73, carotenoids 19.329 ± 3.05, lycopene 1.828 ± 0.05, and anthocyanins 17.397 ± 0.75. In the case of anthocyanins, the results varied from 16.06 to 33.27 mg.100 g-1, showing the lowest content of these varieties Keitt, on the other hand, the resulting values of the content of carotenoids and lycopene were slightly higher in the mentioned variety. For ascorbic acid, the Creole variety stands out, since it showed a higher content.

Then, Ornelas-Paz et al. (2007) indicate that the total content of carotenoids they obtained comprises a range of 1,159 to 3,000 mg/100 g of pulp from different mango cultivars. The extraction of the different compounds mentioned above appears as a potential source for the production of functional foods that improve nutritional content, as is the case of powdered mango peel (Serna Cock and Torres León 2015). Furthermore, *Mangifera indica* has been shown to have antioxidant, antiallergenic, antiatherogenic, anti-inflammatory, antimicrobial, and antithrombotic effects (Alexander et al. 2016). Research carried out by Kim et al. (2012) shows that ethanolic extracts from the Irwin variety of mango peel contribute to the prevention of cervical cancer. Finally, the use of extracts from leaves and stems of this tropical fruit tree has been detailed and exposed in traditional medicine as a palliative for the treatment of dental and muscular pain, certain inflammatory conditions.

*Mangifera indica* allows for a field of study concerning each morphological part that makes it up, since although the presence of different bioactive compounds has been demonstrated in them, it is in the pulp where they are most concentrated; this has in the food industry a wide application in the elaboration of different matrices; however, the presence of bioactive compounds is known in other parts such as seeds, leaves, and shell, which through certain transformations allow them to be used for the improvement of the contents. nutrients in food processing.

### Psidium guajava

*Psidium guajava* known as guava is a native tree to the American tropics. It is part of the *Myrtaceae* family, and is rich in nutrients, especially vitamin A and C, as well as fiber and antioxidants (Barazarte Barazarte et al. 2015). The fruit of this tree has aroused the interest of the agro-industrial sector due to its qualities in taste, appearance, nutrients, and free radical scavenging and anti-inflammatory activity that it promotes to improve health (McCook-Russell et al. 2012), thus being considered a desirable raw material for the manufacture of functional products. Guava is currently used in the production of products such as jams, jellies, juices, concentrates, syrups, and canned and dehydrated products, among others (Jiménez-Escrig et al. 2001), also generating waste that can negatively impact the environment, which is why strategies have been developed to take advantage of these residues in the creation of new products or the extraction of bioactive compounds. However, studies related to the biological activities of its different non-usable morphological parts such as branches, fruits, flowers, and leaves are very scarce.

Ramírez and Pacheco de Delahaye (2011) evaluated the composition of bioactive compounds present in the pulps of several tropical fruits, pineapple, soursop, and guava, and for the latter, which is the one of interest, they obtained 56.93 ± 0.134 mg/100 g of total polyphenols, 28.79 ± 0.134 mg /100 g of total carotenoids and 179.6 ± 0.643 mg/100 g of vitamin C, highlighting its content of antioxidant compounds in guava. In the case of guava leaves, it has been shown to have anti-plaque properties, which is why herbalists recommend its use for the prevention and treatment of oral conditions, also allowing fresh and clean breath (Kaneria and Chanda 2011). The leaves present β-selinene, α-humulene, β-caryophyllene, Quercetin, gallocatechin, esculin, 3-sinapoylquinic acid, ellagic acid, gallic acid, citric acid, hyaluronic acid, chondroitin sulfate and ulvan depending on the extraction techniques, which present antioxidant and antimicrobial activity (de Souza et al. 2021).

## Extraction technologies of bioactive ingredients

The extraction of bioactive compounds from different food matrices of plant origin is carried out using different technologies used in the cosmetic, pharmaceutical and food industries (Rodríguez-Riera et al. 2014), which seek to improve the processes and operations that allow the acquisition of different compounds to ensure their stability and precision. In the application of the different extraction methods, it is essential to control the environmental conditions in which the extraction process is carried out, such as the temperature, the polarity of the solvents used, and the pH of the solution, regardless of the methodology applied (Martínez-Navarrete et al. 2008).

Different extraction methods have been used for the extraction of bioactive ingredients for tropical fruits (Table 1). Methods such as maceration and Soxhlet are conventionally used but have been shown to require large amounts of chemical solvents, in addition to involving external operations for their correct development, which implies the investment of long periods to obtain bioactive compounds (Soquetta et al. 2018), For this reason, friendlier technologies have been implemented to obtain advantages over the applied process, which show better efficiency in the optimal isolation of compounds, keeping the biological activity of each one intact (Bucar et al. 2013) within these we can mention the technique of maceration, ultrasound, and supercritical fluid technology.

**Table 1:** Extraction technologies, bioactive compounds, and biological activities of tropical fruits

Tropical fruits	Source	Technology of extraction	Bioactive compounds	Biological activities	Refs.
*Tamarindus indica*	Seed	Soxhlet	p-Xylene, Hydrazinecarboxylic acid, phenylmethyl est, Benzeneethanol,alpha-, beta-dimethyl-, 1-Decyne, 1-Hexanol, 2-ethyl-, Oxalic acid, isobutyl nonyl ester, p-Cymene, *n*-Hexadecanoic acid, Tritetracontane,	–	Fagbemi et al. (2021)
	Seed	Hidrodistillation	o-Xylene, p-Xylene, 3-tert-Butyl-5-chloro-2-hydroxybenzophenone, Dodecane, 2H-pyran, tetrahydro-4-methyl-2-(2-methyl, cis-3-Methylcyclohexanol, Benzene, 1-ethyl-2,3-dimethy, Undecane, n-Hexadecanoic acid, Phytol, acetate	–	Fagbemi et al. (2021)
	Seed coat	Maceration	Disaccharide, Citric acid, Protocatechuic acid, Procyanidin dimer digallate, Epìcatechin, Rutin, Quercetin-O-hexoside, Oxo-dihydroxy-octadecenoic acid, Trihydroxy-octadecenoic acid	Antioxidant activity	Cvetanović et al. (2020)
	Fruit	Supercritical Fluids Extraction	$\alpha$-pinene, $\beta$-pinene, myrcene, furfural, linalool, carvacrol, $\alpha$-copaene, $\beta$-caryophyllene, terpinene-4-ol, $\alpha$-humulene, $\gamma -$ cadinene, $\beta -$ elemene, octadecane	–	Sagrero-Nieves et al. (2011)
*Annona muricata*	By-products	Ultrasound-Assisted Extraction	Gallic acid, Coumaric acid, Cinnamic acid, Caffeic acid, Chlorogenic acid, Protocateic acid, Hydroxybenzoic acid, Syringic acid, Neochlorogenic acid	–	Aguilar-Hernández et al. (2019)
	Fruits	Solid–liquid extraction	isomers of annonacinone, isomers of gonionenin, isomers of montanacin-D	–	Laboureur et al. (2017)
	Leaves	Soxhlet	12,15-cis-squamostatin-A, squamostatin-A; bullatacin; Squamocin; Isodesacetyluvaricin; Desacetyluvarici	Antioxidant	Nik Mat Daud et al. (2015)
*Mangifera indica*	Peel	Ultrasound-Assisted Extraction	–	Antioxidant	Martínez-Ramos et al. (2020)
	flower	Microwave-Assisted Extraction /hydrodistillation	$\alpha$-pinene, camphene, sabinene, myrecene, $\beta$-pinene, d-3carene, $\alpha$-terpinene, limonene, terpinoele, lilaool	–	Wang et al. (2010)
	Peel	Supercritical fluids extraction	Mangiferin, gallic acid, ellagic acid, quercetin	–	Sánchez-Mesa et al. (2020)
	By-products	ultrasound-assisted extraction	Mangiferin	Antioxidant	Borrás-Enríquez et al. (2021)
*Psidium guajava*	Leaves	Steam distillation	β-selinene, α-humulene, and β-caryophyllene	Antioxidant/Antimicrobial	de Souza et al. (2021)
	Leaves	Solid–liquid extraction	Quercetin, gallocatechin, esculin, 3-sinapoylquinic acid, ellagic acid, gallic acid, citric acid	Antibacterial	Sampath Kumar et al. (2021)
	Leaves	Ultrasound assisted extraction	Hyaluronic acid, chondroitin sulfate, ulvan	Antioxidant	Alves Amaral et al. (2020)

The maceration method is used in the extraction of bioactive materials that belong to a solid that is brought to the liquid state. It is considered that in the solid state the product contains some compounds that are soluble in the liquid used as a solvent to extract the desired bioactive compound. This process requires time that sometimes exceeds days, and it is necessary to control external factors such as light and temperature to obtain favorable results (Capasso et al. 2011). Furthermore, it is essential to take into account the nature of the solid product, the types of solvent used, and the chemical nature of the compounds that must be extracted. This technique has been used to obtain *Tamarindus indica*. Díaz-Torres (2017) determined the antioxidant capacity of extracts obtained from the leaves of mango var. Tommy Atkins, Edwar and Haden. Although this traditional technique has certain advantages, such as the low cost of assembling the extractor unit, it shows certain disadvantages in its application, within which the time spent is evident, since for its development and obtaining results, it is required several days, without counting the excessive amount of solvents used, which makes it an inefficient method.

The Soxhlet technique that performs an exhaustive extraction of the soluble components in the hot solvent, in addition, for the use of this technique a large amount of solvents is not required and favorable results are obtained (Bourdon-García 2017). This method was applied in the study by Vitola et al. (2016) where they analyzed the bioactivity of the *Annona muricata* extract and the essential oils of *Citrus aurantium* against *Phytophthora cinnamomic*.

Ultrasonically assisted extraction turns out to be an attractive technique as it is environmentally friendly, requires short processing times, and does not require the use of large amounts of solvent. The ultrasound process is carried out through the phenomenon of cavitation, which causes material rupture caused by waves in the cell wall, giving way to the release of compounds; This allows the solvent to heat up, thus increasing the diffusion of the extract and therefore improving mass transfer through the solid–liquid interface (Medina-Torres et al. 2017). This method can be carried out directly, using a system of probes that are introduced into the solid–liquid interface with a high force; and indirectly, using an ultrasonic water bath, in which the sample is introduced into a balloon with the solvent and the waves travel through the water to the plant material (Wong-Paz et al. 2020).

Different studies have been carried out to obtain bioactive compounds from tropical fruits. Aguilar-Hernández et al. (2019) obtain an extract from *Annona muricata* by-products, Martínez-Ramos et al. (2020) obtain antioxidant fractions from *Mangifera indica*, and Alves Amaral et al. (2020) obtain antioxidant extracts from *Psidium guajava* leaves.

Extraction assisted by supercritical fluids is within the group of green technologies since it facilitates a rapid extraction using moderate temperatures, thus allowing the degradation of the desired compounds not to occur, this technique excludes within its methodology previous washing stages and does not require the use of toxic solvents in the process, so it is friendly to the environment since it uses CO_2_, which makes this an extraction method with many advantages, among them the increase obtained from the yields of compounds of interest. The extraction of bioactive compounds by this method consists of several phases, beginning with pressurization, where the pressure is raised beyond the critical pressure of the solvent, and then a temperature adjustment is made, lowering or raising it until the solvent obtains the temperature required for the extraction, taking into account that it must be above its critical temperature; in this way, the extraction is carried out, where the supercritical fluid interacts with the solute in the extractor, to later decompress the solvent at a pressure less than critical and to achieve as a result the release of the solute. Different authors employed the supercritical fluid extraction for the extraction of extracts or oil from tropical fruits: Mokbul et al. (2022) extract supercritical mango kernel extracts with palmitic acid (C16), stearic acid (C18), oleic acid (C18: 1) and linoleic acid (C18:2); Dorado et al. (2016) applied in the extraction of oil present in the seeds of soursop (*Annona muricata*) with supercritical CO_2_, obtaining samples with values between 1.40 and 2.81 mg/ml of total sterols, identifying β-sitosterol with an average concentration of 10.02 mg/ml; also, stigmasterol showed a concentration of 0.73 mg/ml and campesterol with a concentration of 0.36 mg/ml, with β-sitosterol being the compound that showed the highest presence in the seed oil of soursop; Idris et al. (2022) Obtain tamarind seed oil with an extraction yield between 0.04 to 0.34 wt% exhibited a unique phenolic and miscellaneous compound profile, a high proportion of β-sitosterol compared to that of the other phytosterols, a high contribution of γ-tocopherol to the tocopherol composition, a high contribution of linoleic acid to the fatty acid profile, and low stability to lipid oxidation, the oil present arachidic acid (35.2 mol%), α-linolenic acid (17.4 mol%), behenic acid (8.7%), gadoleic acid (7.4%), γ-linolenic acid (5.7%), tridecylic acid (4.7%), palmitoleic acid (4.4%), margaric acid (4.0%), stearic acid (3.7%), lauric acid (3.4%), myristic acid (2.7%), and oleic acid (2.7%).

Sagrero-Nieves et al. (1994) obtain the volatile component from the fruit of *Tamarindus indica* L with 3% of furfural, 0.10% of limalool, 0.50% carvacrol, 0.6% of $\alpha$-copaenene, $\beta$-caryophyllene, 90% of aromadendrene, 2% of $\alpha$-humulene, 0.4% of $\gamma$-cadinene, 0.20% of $\alpha$-muurolene, 0.30% $\beta$-elemene and 0.15% of octadecane; Narváez-Cuenca et al. (2020) obtain oil form guava seed with extraction yield of 8.6 ± 1.2 g oil/100 g of seed similar (no significant different, p > 0.05) to that obtained when the extraction was performed by the Soxhlet method (9.1 ± 0.3 g oil/100 g guava seeds); and with 10.6% of palmitic acid, 3.2% of stearic acid, 7.7 of oleic acid and 78.5% of linoleic acid; and Moura et al. (2012) obtain guava leaf extract with higher level of functional compound in comparison with Soxhlet extraction, such as Essential oils, flavonoids, carotenoids and anti-oxidant compounds. Then, supercritical fluid extraction is an alternative for the extraction of bioactive compounds.

## Conclusion

The bioactive compounds present in tropical fruits such as *Tamarindus indica L., Annona muricata, Mangifera indica, and Psidium guajava*, determine them as a good source of bioactive compounds, the most abundant being carotenoids, phenolic compounds, ascorbic acid, anthocyanins, and lycopene, which are distributed in the different morphological parts of the tropical fruits mentioned, such as leaves, seeds, peels and pulp. In general, *Tamarindus indica L.* extracts present volatile compounds such as $\alpha$-pinene, $\beta$-pinene, myrcene, furfural, linalool, carvacrol, $\alpha$-copaene, $\beta$-caryophyllene, terpinene-4-ol, $\alpha$-humulene, $\gamma -$ cadinene, octadecane, *Annona muricata* extracts vitamins C and E, carotenoids, anthocyanins, flavonoids, *Mangifera indica* extracts anthocyanins, carotenoids, lycopene 1and ascorbic acid and *Psidium* guajava extracts has β-selinene, α-humulene, β-caryophyllene, Quercetin, gallocatechin, esculin, 3-sinapoylquinic acid, ellagic acid, gallic acid, citric acid and hyaluronic acid. The biological activities of these compounds are of great importance in human health due to their positive impact on it and favor the prevention and treatment of dental and muscular pain, chronic degenerative diseases, inflammatory diseases, and cancer, due to the antioxidant power conferred by bioactive compounds on tropical fruits. The data obtained show the need to implement the operational processes that allow the different compounds to be obtained, which ensure their stability and precision, applying different extraction methods, such as maceration, Soxhlet, supercritical fluids, and ultrasound, for the development of food ingredients from tropical fruits. Therefore, the importance of the information contained in this document is ratified, since it allows one to have a clear idea about the use of these resources, and their benefits to improve the health of human beings, as would be the case of their application in the development. of products that provide a beneficial effect on health, such as nutraceuticals or functional food ingredients.
